# The sugar donor specificity of plant family 1 glycosyltransferases

**DOI:** 10.3389/fbioe.2024.1396268

**Published:** 2024-05-02

**Authors:** Hani Gharabli, Ditte Hededam Welner

**Affiliations:** The Novo Nordisk Center for Biosustainability, Technical University of Denmark, Kongens Lyngby, Denmark

**Keywords:** glycosyltransferase, donor specificity, enzyme engineering, glycodiversification, structure-function

## Abstract

Plant family 1 glycosyltransferases (UGTs) represent a formidable tool to produce valuable natural and novel glycosides. Their regio- and stereo-specific one-step glycosylation mechanism along with their inherent wide acceptor scope are desirable traits in biotechnology. However, their donor scope and specificity are not well understood. Since different sugars have different properties *in vivo* and *in vitro*, the ability to easily glycodiversify target acceptors is desired, and this depends on our improved understanding of the donor binding site. In the aim to unlock the full potential of UGTs, studies have attempted to elucidate the structure-function relationship governing their donor specificity. These efforts have revealed a complex phenomenon, and general principles valid for multiple enzymes are elusive. Here, we review the studies of UGT donor specificity, and attempt to group the information into key concepts which can help shape future research. We zoom in on the family-defining PSPG motif, on two loop residues reported to interact with the C6 position of the sugar, and on the role of active site arginines in donor specificity. We continue to discuss attempts to alter and expand the donor specificity by enzyme engineering, and finally discuss future research directions.

## 1 Introduction

Glycosides are abundant in the biosphere and are represented by numerous high-value industrial compounds. This includes natural sweeteners such as steviol glycosides (Libik-Konieczny et al., 2021) and mogrosides (Li et al., 2022), therapeutic compounds such as amygdalin (Thodberg et al., 2018) and glycyrrhizin (Li et al., 2010), and natural colourants such as betanin (Zhang L. et al., 2023). The current or potential high demand for these compounds and other glycosides requires their efficient production (Nidetzky, 2021). Since glycosylation using conventional chemistry is notoriously challenging and usually inefficient, attention has been turned to biocatalysts for the glycosylation of organic compounds (De Roode et al., 2003). Here, family 1 glycosyltransferases (Drula et al., 2022) are in focus due to their ability to regio- and stereoselectively transfer a sugar molecule from a uridine diphosphate (UDP)-activated donor to a wide variety of acceptors in one step (Yao et al., 2022). These enzymes are also commonly referred to as UDP-dependent glycosyltransferases (UGTs). The catalytic cavity of UGTs is known to accommodate a varied panel of acceptors while still retaining catalytic activity, making them suitable for the synthesis of *new-to-nature* glycosides (De Bruyn et al., 2015).

**SCHEME 1:**
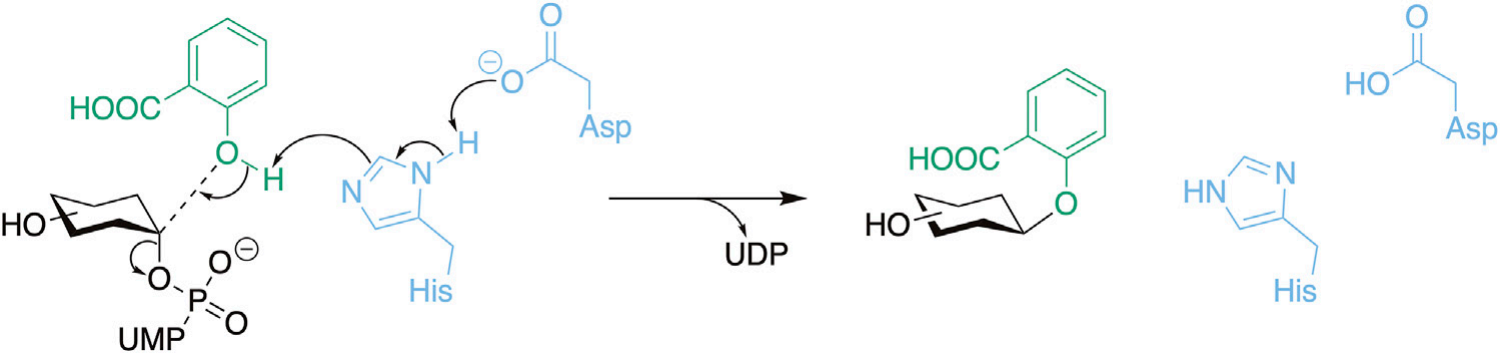
The general reaction mechanism of UGTs with the catalytic residues presented in blue, the donor substrate in black, and the acceptor substrate (salicylic acid) in green. Here, *O*-glycosylation is used as an example which includes a deprotonation step.

UGTs have been extensively studied, which has led to a broad understanding of their catalytic mechanism and acceptor specificity (Franconetti et al., 2021; Teze et al., 2021). Their mechanism involves a highly conserved catalytic dyad, typically comprising His and Asp. In *O*-glycosylation, this dyad deprotonates the acceptor, facilitating nucleophilic attack (Scheme 1) (Brazier-Hicks et al., 2007; Teze et al., 2021). However, *O*-/*N*-/*S*-glycosylation all occur via an SN2-like reaction and inversion of the anomeric configuration (Scheme 1) (Teze et al., 2021). This knowledge has yielded several successful engineering efforts leading to enhanced catalytic efficiency (e.g., Li et al., 2020), altered regiospecificity (e.g., Li et al., 2021; Zhang M. et al., 2022), and altered acceptor substrate scope (e.g., Wetterhorn et al., 2017). However, the structure-function relationship determining the donor specificity of the enzyme family is still poorly understood. UGTs adopt a GT-B fold consisting of two Rossmann-type fold domains where the acceptor predominantly interacts with the N-terminal domain while the donor substrate predominantly interacts with the C-terminal domain (Wang M. et al., 2023). A highly conserved motif resides within the C-terminal domain, denoted as the Plant Secondary Product Glycosyltransferase (PSPG) motif (Hughes and Hughes, 1994; Paquette et al., 2003). This motif is recognised as a critical part of donor binding (Li et al., 2007) and has laid grounds for several mutational studies with mixed results (*vide infra*). However, residues outside of the PSPG motif and from the N-terminal domain have also been demonstrated to be important for the donor specificity, illustrating the complexity of this structure-function relationship (Osmani et al., 2009).

**FIGURE 1:**
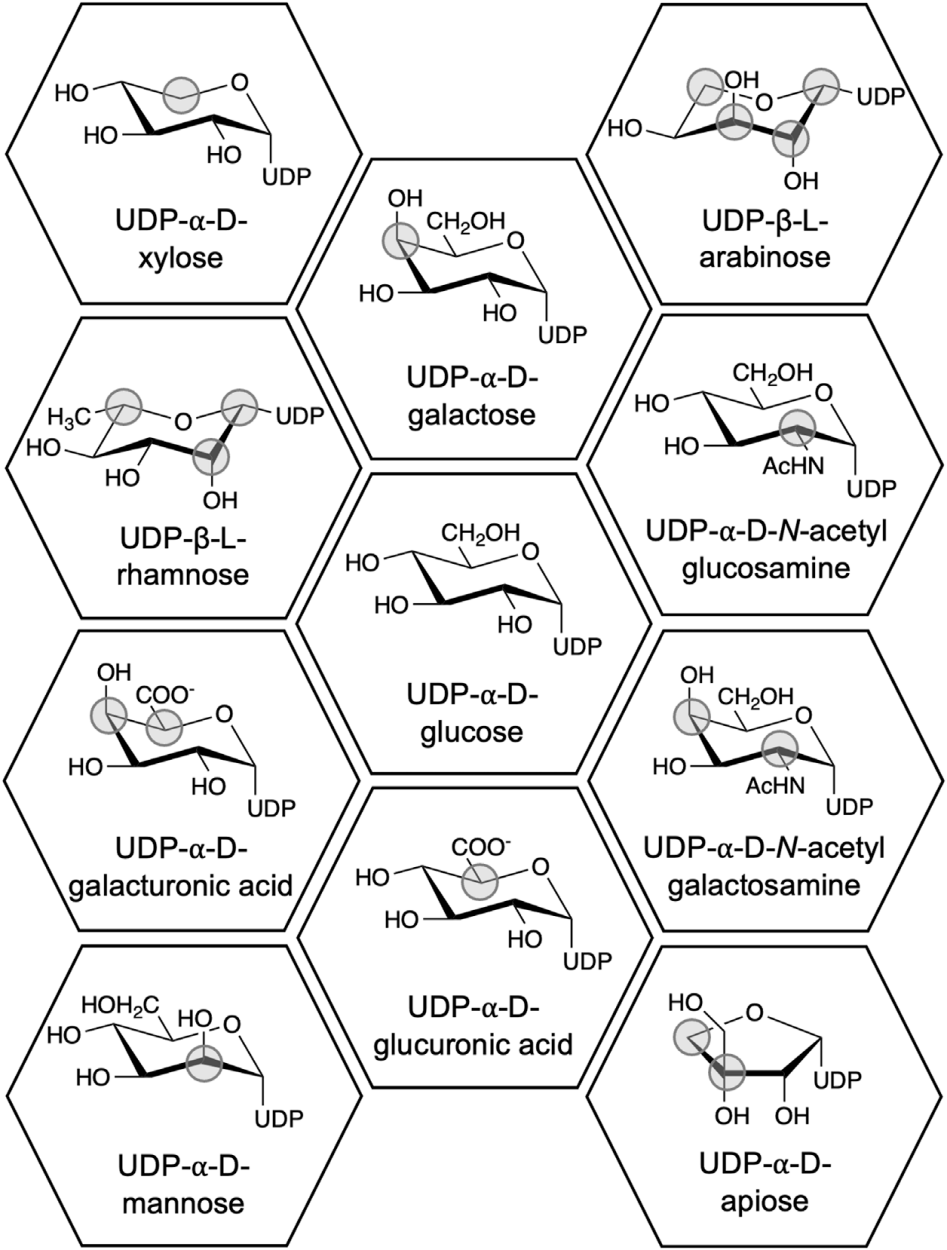
Chemical structures of UDP-sugars which have been discovered to be donor substrates for UGTs. The differences between the UDP-sugars have been marked with a grey circle using UDP-Glc as the reference structure. UDP-apiose was compared to the Haworth projection of UDP-α-d-glucofuranose.

UGTs from plants most often use UDP-glucose (UDP-Glc) as donor (Louveau and Osbourn, 2019). However, UDP-galactose (UDP-Gal), -glucuronic acid (-Glu), and -rhamnose (-Rha) are also known plant UGT substrates (Figure 1). These sugars can potentially provide specific properties to an organic molecule upon conjugation (Goel et al., 2021; Zhang L. J. et al., 2022), but understanding these properties is hindered by the narrow donor substrate scope of UGTs, which usually includes only one UDP-sugar (Osmani et al., 2008). Hence, understanding the structure-function relationship of UDP-sugar specificity is a coveted goal in the research field. Despite numerous successful alterations of donor specificity (e.g., Liu S. et al., 2021; Chen et al., 2021; Huang et al., 2022), the structure-function relationship is still not well-defined (Rahimi et al., 2019). Instead, we now have an extensive knowledge base of the effects of mutating specific residues in specific UGTs. In addition, several high-resolution structures of UGTs in complex with UDP-sugars have been solved (Table 1).

**TABLE 1:** Reported crystal structures of UGT:UDP-sugar complexes.

UGT isoform	Plant species	UDP-sugar	Resolution (Å)	Crystallisation method	PDB	Reference
**UGT71G1**	*Medicago truncatula*	UDP-Glc	2.60	Soaking	2ACW	Shao et al. (2005)
**UGT708C1**	*Fagopyrum esculentum*	UDP-Glc	2.01	Co-crystallisation	6LLZ	Liu et al. (2020a)
**UGT74AC1**	*Siraitia grosvenorii*	UDP-Glc	2.10	Soaking	6L8Z	Li et al. (2020)
***Sb*CGTb**	*Scutellaria baicalensis*	UDP-Glc	2.87	Co-crystallisation	6LFZ	Wang et al. (2020)
***Pt*UGT1**	*Polygonum tinctorium*	UDP-Glc	2.40	Soaking	6SU6	Teze et al. (2021)
***Mi*CGT**	*Mangifera indica*	UDP-Glc	2.85	Co-crystallisation	7VA8	Wen et al. (2021)
**UGT74AN2**	*Calotropis gigantea*	UDP-Glc	2.15	Soaking	7W1H	Huang et al. (2022)
***Gg*CGT**	*Glycyrrhiza glabra*	UDP-Glc	2.60	Co-crystallisation	6L5P	Zhang et al. (2020)
***Gg*CGT**	*Glycyrrhiza glabra*	UDP-Gal	2.89	Co-crystallisation	6L5Q	Zhang et al. (2020)
**UGT89C1**	*Arabidopsis thaliana*	UDP-Rha	3.21	Co-crystallisation	6IJA	Zong et al. (2019)
**UGT76G1**	*Stevia rebaudiana*	UDP-Xyl	2.50	Co-crystallisation	6KVJ	Liu et al. (2020b)

To foster additional research into UGT donor specificity, this review aims to delineate and establish connections within the existing knowledge base. Furthermore, the research direction will be discussed and related to future biocatalytic research.

## 2 The role of the PSPG motif in the donor specificity of UGTs

**FIGURE 2:**
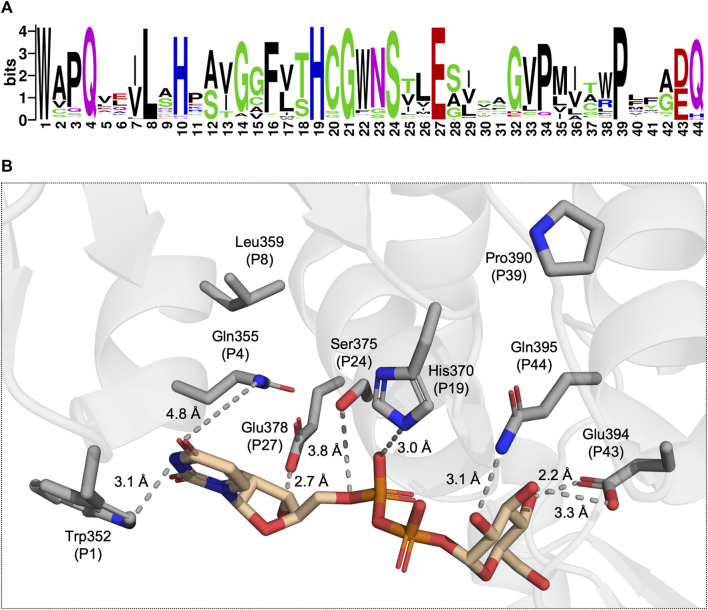
**(A)** A sequence logo generated from a multiple sequence alignment of 224 UGTs, each with determined donor specificity. **(B)** A view of the donor substrate binding site with the conserved residues of the PSPG motif displayed as grey sticks and UDP-Glc displayed in tan (PDB: 6SU6). The dotted lines represent potential intermolecular interactions between the UGT and UDP-Glc.

The PSPG motif is a key characteristic of plant UGTs and is commonly used in genome mining to identify UGTs (Fan et al., 2017; Rehman et al., 2018). The highly conserved motif is constituted by 44 residues with Trp as the initial residue and an acidic residue followed by Gln/His/Asn as the terminal residue pair. The PSPG motif contains several highly conserved positions, namely, positions 4, 8, 19, 21, 24, 27, and 39 (Figure 2A). Observing these residues in a crystal structure reveals that all, except Leu P8 and Pro P39, can participate in intermolecular interactions with the UDP moiety of the UDP-sugar (Figure 2B). Meanwhile, among the conserved residues within the PSPG motif, only P43 and P44 can interact with the sugar moiety (Figure 2B). Given conservation of the PSPG motif along with its role in UDP-sugar recognition, studies have targeted the motif to alter the donor specificity of UGTs.

Due to its direct interaction with the sugar moiety and high conservation, P44 of the PSPG motif has often been the target of site-directed mutagenesis (e.g., Zong et al., 2019; Yamashita et al., 2023). Noticeably, the bifunctional glucosyl-/galactosyltransferase VvGT6 from *Vitis vinifera* was converted into a monofunctional galactosyltransferase by a single substitution of Gln373 with His (Ono et al., 2010a). However, the substitution of this Gln with His in the glucosyltransferases, UBGT, VvGT1, MrUFGT, and MrUGT72B67, did not confer UDP-Gal activity (Kubo et al., 2004; Offen et al., 2006; Ren et al., 2022a). Furthermore, wildtype galactosyltransferases with Gln or Asn at P44 are known (Xie et al., 2020; 2022; Ren et al., 2022b). Even though it is not possible to directly correlate the residue at P44 with UDP-Gal activity, many studies have demonstrated the importance of this residue in UDP-sugar specificity. Particularly mutating P44 into Gln has led to interesting discoveries. In the galactosyltransferase, *Ac*GaT from *Aralia cordata,* His to Gln substitution at P44 improved UDP-Glc activity (Kubo et al., 2004). Similarly, changing His to Gln in the UDP-Rha-specific UGT89C1 led to an increase in the UDP-Rha activity while also enabling activity with UDP-Glc (Zong et al., 2019). In the arabinosyltransferases *As*AAT1 from *Avena strigose* and UGT78D3 from *A. thaliana,* swapping P44 from His to Gln improved or enabled activity with UDP-Glc and UDP-Xyl while it significantly reduced UDP-Ara activity in the case of *As*AAT1 (not tested for UGT78D3) (Han et al., 2014; Louveau et al., 2018).

PSPG positions other than P44 have also been mutated to understand their function. The glucosyltransferase UGT76E2 from *A. thaliana* was converted into a bifunctional UGT accepting both UDP-Glc and UDP-Gal (Akere et al., 2020). This was facilitated by mutating Asp374 (P43) to Glu, where the extra carbon atom enabled hydrogen bonding between Glu and the C4-OH of galactose. Meanwhile, mutating P43 to Ala has in several instances been shown to abolish glycosyltransferase activity, suggesting the importance of this residue, the acidity of which might be important for UDP-sugar recognition (Shao et al., 2005; Offen et al., 2006; Zong et al., 2019; Zhang S. et al., 2023). Another study expanded the donor specificity of UGT78H2 by mutating P3 and P23 (Chen et al., 2021). Wildtype UGT78H2 accepted UDP-Glu and -Gal while the introduction of mutations Asn340Pro (P3) and Lys360Asn (P23) expanded the donor scope to include UDP-Glc, and in addition improved the catalytic efficiency with UDP-Glu and -Gal by 7% and 30%, respectively. Molecular docking with UDP-Gal and -Glu showed that Asn in P23 produced an additional intermolecular interaction with the uridine moiety and a direct interaction with galactose as compared with Lys. Meanwhile, Liu S. et al., 2021 were able to increase the UDP-Glc activity in the galactosyltransferase, *Tc*OGT4, by swapping P22 of the PSPG from Pro to Trp. This residue was identified through multiple sequence alignment with other glucosyltransferases. In addition to these examples, other studies have successfully altered the UDP-sugar donor specificity of UGTs by mutating residues in the PSPG motif. These are related to topics which are discussed in sections 4 and 5 and are accordingly referenced within those sections.

Overall, the PSPG motif naturally serves as an integral part of donor substrate recognition. However, it is limited how much information can be extracted from the constituents of the motif and related to the donor specificity of a UGT. Many efforts have targeted the terminal residue with mixed results. Meanwhile, targeting other parts of the PSPG motif has proven an interesting path to altering donor specificity even though they, most of the time, do not interact with the sugar moiety, indicating a role in the orientation of the UDP-sugar.

## 3 A loop in the N-terminal domain can significantly alter activity with UDP-Rha, -Glc, and -Xyl

Rhamnose differs from the previously mentioned sugars by not containing a hydroxy group on C6. In addition, UDP-Rha naturally occurs in the β-l configuration, where the other substrates are in the α-d configuration (Figure 1), which impacts the overall geometry of UDP-Rha. These characteristics need to be accommodated to enable activity with UDP-Rha.

**FIGURE 3:**
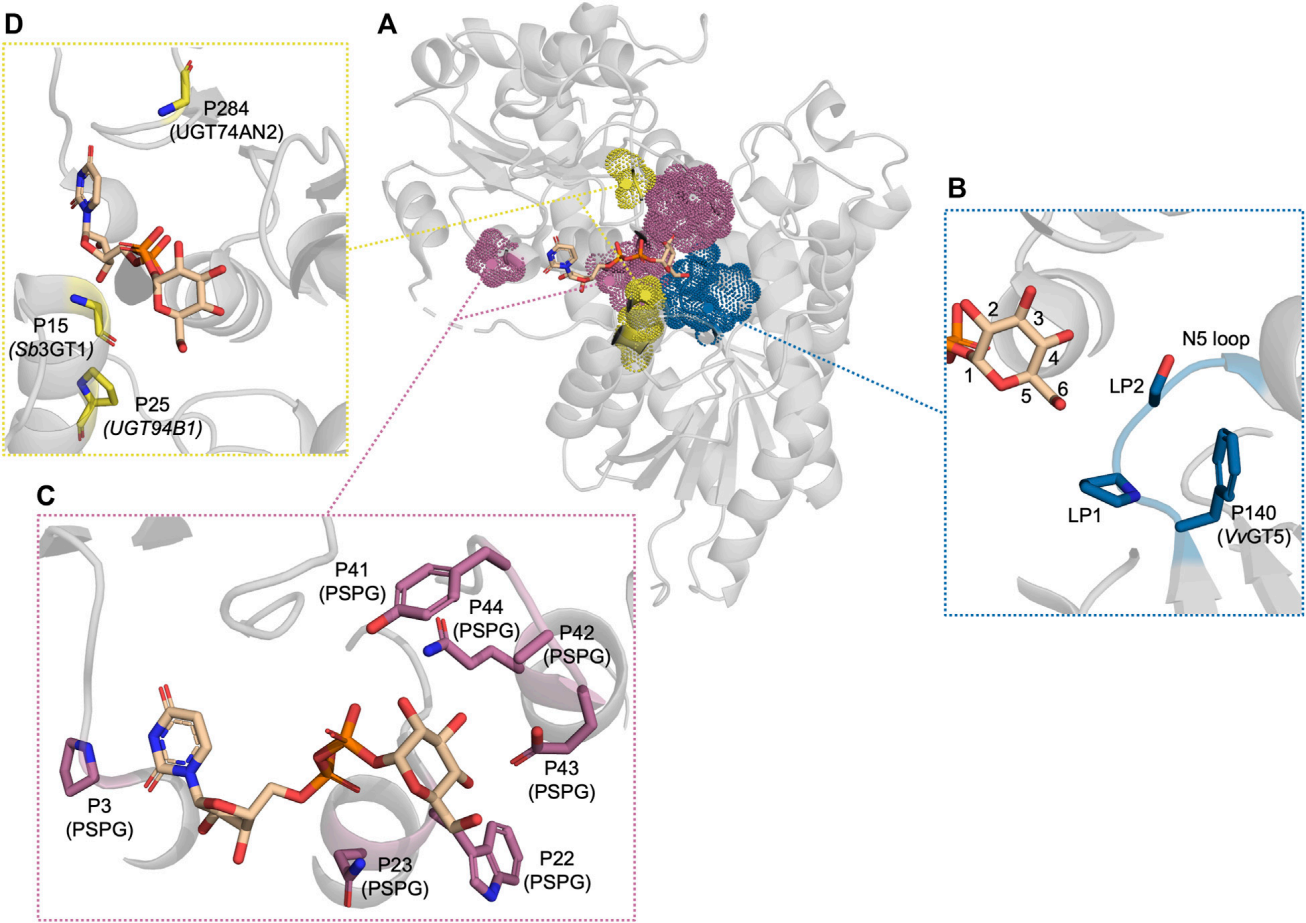
The residues which have been reported to be important for UDP-sugar donor specificity, displayed in PDB: 6SU6. **(A)** An overview of the residues displayed as dot spheres representing the van der Waals surface area. Each color corresponds to a specific area: blue indicates the N5 loop, magenta indicates the PSPG motif, and yellow indicates residues near the cavity entrance. **(B)** A close-up view of the N5 loop area. **(C)** A close-up view of the PSPG motif area. **(D)** A close-up view of the residues close to the cavity entrance. “P” refers to the position in the corresponding UGT or PSPG motif mentioned in parentheses.

Several studies have focused on residues in proximity to C6 to understand how the activity with UDP-Rha can be enabled and disabled. In the UDP-Rha-bound crystal structure of the rhamnosyltransferase, UGT89C1 (Table 1), Pro147 (loop position 1 (LP1)) and Ile148 (LP2) were found to be in the vicinity of C6 (Figure 3B) (Zong et al., 2019). These two residues reside in a flexible loop, which was first described as an important loop for donor specificity by Osmani et al., 2009 and dubbed the N5 loop (Figure 3B). Swapping LP1 in UGT89C1 for the corresponding residue in the UDP-Glc-specific UGT71G1 (Thr) abolished rhamnosyltransferase activity. Meanwhile, swapping LP2 for the corresponding residue in UGT71G1 (Ser) reduced the activity to 20% relative to WT. Similar observations were made in a study of two UGTs from *Viola tricolor*, VtCGTa and VtCGTc, which displayed a preference for UDP-Glc and UDP-Rha, respectively (Han et al., 2023). Aligning VtCGTc and other rhamnosyltransferases with VtCGTa and other glucosyltransferases showed that a Thr occupied LP1 in the glucosyltransferases while Val, Pro, or Ile occupied this position in the rhamnosyltransferases. Mutating this position in VtCGTa its homologs to Val or Pro improved UDP-Rha activity in all instances while mutating the position into Ile only enabled UDP-Rha activity in some of the tested CGTa enzymes. Mutating LP1 in VtCGTc from Val to Thr significantly reduced the UDP-Rha activity and established activity with UDP-Glc. These results indicate that a hydrophobic residue in LP1 could be an important factor for UDP-Rha activity. However, these effects were not observed in the rhamnosyltransferase, *Hm*F3RT, which also has activity with UDP-Xyl, and UDP-Glc (Zhang S. et al., 2023). Mutating Val129 (LP1) to Ala or Thr, improved activity with UDP-Rha and significantly improved the UDP-Glc activity to a level comparable to the UDP-Rha activity, while it decreased the activity with UDP-Xyl.

UDP-Glc and -Xyl activity has been demonstrated to be susceptible to changes when altering LP1 and LP2. One study identified two UGT89A2 homologues from 2 *A. thaliana* accessions which had distinct donor specificities (Chen and Li, 2017). UGT89A2 from Col-0 accepted UDP-Xyl and harboured an Ile at LP2 while UGT89A2 from C24 accepted UDP-Glc and UDP-Xyl and harboured a Ser at LP2. Swapping LP2 residues also led to a swap in donor specificity. A similar method was employed with Ib3GGT from *Ipomoea batatas*, which accepted UDP-Glc and UDP-Xyl as donor substrate, and its homologue from *A. thaliana*, At3GGT, which solely accepted UDP-Xyl (Wang et al., 2018). Scanning the donor binding pocket led to the identification of LP1 as the only variable residue, where Ib3GGT has a Thr while At3GGT has an Ile. Swapping the residues in the two UGTs also swapped their respective donor specificities. Other examples are described in Table 2.

**TABLE 2:** Site-directed mutagenesis of LP1/LP2 and the change in donor specificity compared to WT *VtCGTa T145P was only tested with UDP-Rha and *Vt*CGTc was only tested with UDP-Glc and UDP-Rha.

UGT isoform	Plant species	LP1/LP2 (WT)	Donor specificity (WT)	LP1/LP2 (mutant)	Donor specificity (mutant)	Reference
**UGT89A2**	*Arabidopsis thaliana (Col-0)*	Ser/Ile	UDP-Xyl	Ser/Ser	UDP-Glc/UDP-Xyl	Chen and Li (2017)
**UGT89A2**	*Arabidopsis thaliana (C24)*	Ser/Ser	UDP-Glc/UDP-Xyl	Ser/Ile	UDP-Xyl	Chen and Li (2017)
***Ib*3GGT**	*Ipomoea batatas*	Thr/Ile	UDP-Glc/UDP-Xyl	Ile/Ile	UDP-Xyl	Wang et al. (2018)
***At*3GGT**	*Arabidopsis thaliana*	Ile/Val	UDP-Xyl	Thr/Val	UDP-Glc/UDP-Xyl	Wang et al. (2018)
***Cs*GT2**	*Camellia sinensis*	Ile/Thr	UDP-Glc/UDP-Xyl	Ser/Thr	UDP-Glc	Ohgami et al. (2015)
**UGT74AN3**	*Catharanthus roseus*	Thr/Asn	UDP-Glc	Val/Asn	UDP-Xyl	Huang et al. (2023)
**UGT73F4**	*Glycine max*	Ser/Tyr	UDP-Xyl	Gly/Tyr	UDP-Glc/UDP-Xyl/UDP-Gal	Sayama et al. (2012)
**UGT73F2**	*Glycine max*	Gly/Phe	UDP-Glc	Ser/Tyr	UDP-Xyl	Sayama et al. (2012)
**UGT78W1**	*Morella rubra*	Val/Ser	UDP-Gal	Val/Ala	UDP-Glc/UDP-Gal	Ren et al. (2022b)
**UGT76D1**	*Arabidopsis thaliana*	Pro/Ser	UDP-Glc	Thr/Ser	UDP-Glc/UDP-Gal	Akere et al. (2020)
***As*AAT1**	*Avena strigosa*	Pro/Met	UDP-Ara/UDP-Xyl	Ser/Met	UDP-Ara/UDP-Gal	Louveau et al. (2018)
***Vt*CGTa**	*Viola tricolor*	Thr/Ser	UDP-Glc/UDP-Xyl/UDP-Gal	Pro/Ser	UDP-Rha*	Han et al. (2023)
***Vt*CGTc**	*Viola tricolor*	Val/Ser	UDP-Glc/UDP-Xyl/UDP-Rha/UDP-Ara	Thr/Ser	UDP-Glc/UDP-Rha*	Han et al. (2023)

Recently, a study identified a donor substrate-promiscuous UGT, UGT74AN3, with a preference for UDP-Glc (Huang et al., 2023). This preference was changed to UDP-Xyl and the UDP-Glc activity was significantly reduced upon mutating LP1 from Thr to Val. A molecular dynamics simulation showed that Val could form a hydrophobic interaction with C5 of xylose. Along the same lines, other studies which alter the preference between UDP-Glc and -Xyl make the same argument based on structural modelling and docking, that a hydrophobic residue in LP1/LP2 would clash with C6-OH of UDP-Glc and potentially interact with C5 of xylose, while Ser or Thr would enable the possibility of hydrogen bonding with the hydroxyl group of glucose (Ohgami et al., 2015; Wang et al., 2018). However, when looking at the gathered examples of mutating LP1/LP2 and the alteration in donor specificity (Table 2), it is difficult to identify a simple relationship between the residues of LP1/LP2 and donor specificity. Additionally, reports of enabled UDP-Gal activity upon mutating LP1 have also been reported (Table 2) (Louveau et al., 2018; Akere et al., 2020). In both cases, the WT harbours a Pro at LP1 while mutating LP1 to a hydrophilic residue, Thr or Ser, enabled UDP-Gal activity. This could indicate that the flexibility of the loop plays a significant role, since Pro would rigidify the loop while Thr or Ser would both enhance flexibility and enable potential hydrogen bonding. Finally, newly discovered apiosyltransferases were also found to be specific to UDP-apiose partly via bulky hydrophobic residue on LP1 and LP2, which shows the importance of this loop in the binding of furanoses as well (Wang H. T. et al., 2023; Yamashita et al., 2023).

Altogether, LP1 and LP2 in this N-terminal loop can be considered a hot spot for governing the donor specificity of UGTs. The flexibility along with the hydrophilicity/hydrophobicity of the loop seem to be the most important factors for the donor specificity of UGTs in this region. Lastly, it is worth noting that a loop very similar to the UGT N5 loop is observed in prokaryotic GT-B fold GTs, where structural and mutational studies indicate similar importance in the donor specificity arguing for evolutionary conservation and potentially functional importance (Liu B. et al., 2021; Vuksanovic et al., 2024).

## 4 The role of an active site-arginine in UDP-glucuronic acid activity

**FIGURE 4:**
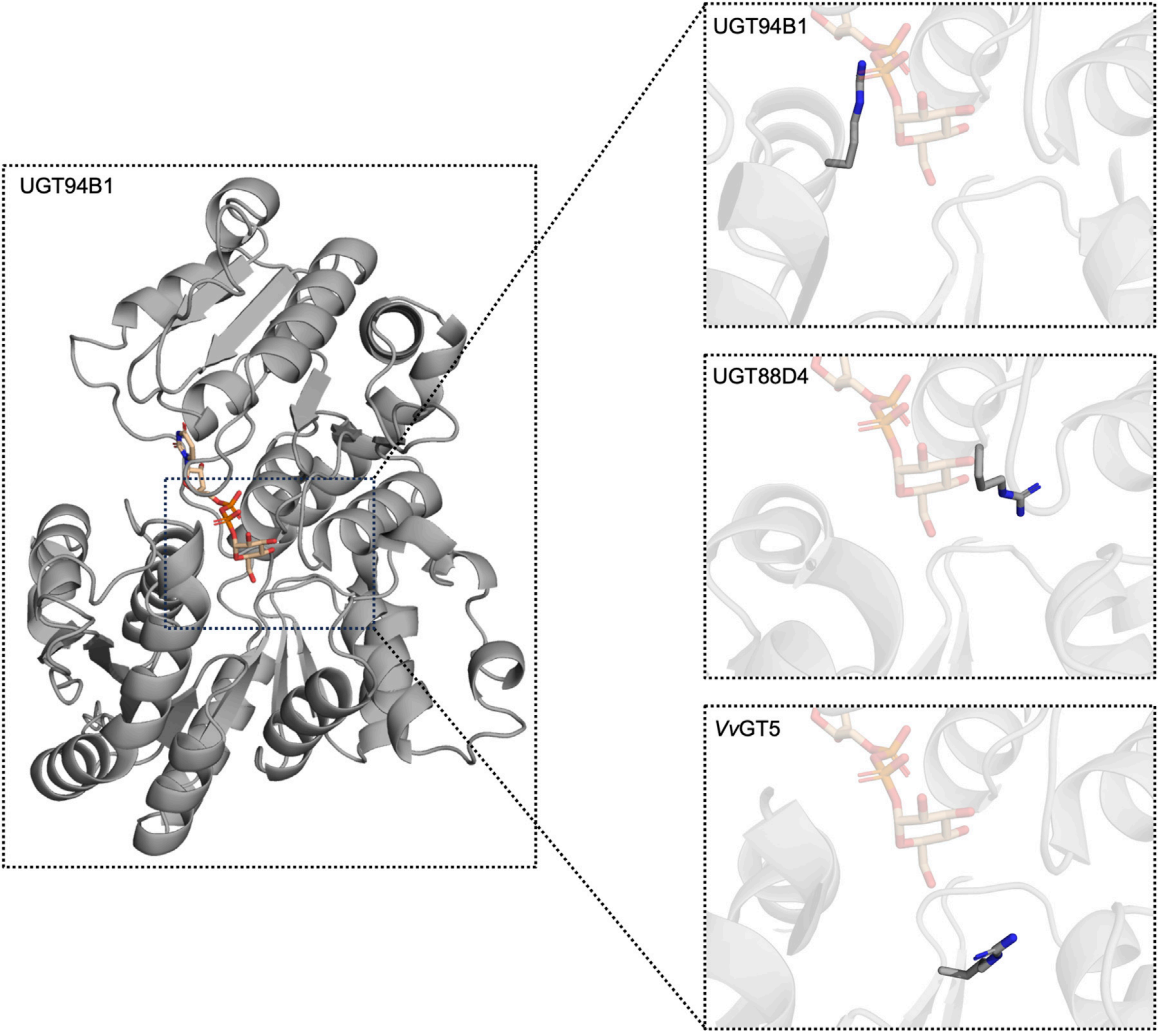
A view of the three different Arg positions described in the text. Alphafold (Jumper et al., 2021) models of UGT94B1, UGT88D4, and VvGT5 were used and had UDP-Glc from PDB 6SU6, displayed in tan, superimposed into the models.

Glucuronosyltransferases play a vital role in the phase II metabolism of exogenous compounds in mammals (Mazerska et al., 2016). In addition, natural glucuronides are found in plants where some isolated glucuronides such as scutallerin-7-*O*-glucuronide (Guo et al., 2014) and apigenin-7-*O*-glucuronide (Ma et al., 2017; Yue et al., 2019) display pharmacological activity. Even though there is a wide variety of glucuronides identified in plants (Yue et al., 2019), only a few plant glucuronosyltransferases have been discovered and characterised. One of these is UGT94B1 from *Bellis perennis*. (Sawada et al., 2005; Osmani et al., 2008). Mutating Arg25 (Figure 4) to either Ser, Gly, Pro, or even another basic residue, Lys, significantly decreased the activity with UDP-Glu, while activity with UDP-Glc was improved in all mutants except Arg25Pro (Osmani et al., 2008). Low UDP-Gal activity was also observed, compared to none with WT. The corresponding Arg in UGT73P12 (Arg32) from *Glycyrrhiza uralensis* was also demonstrated to be important for glucuronosyltransferase activity (Nomura et al., 2019). Mutating residue 32 from Arg to Ser led to a significant decrease in the activity with UDP-Glu while UDP-Glc and -Gal activity was increased 7-fold and 73-fold, respectively.

Even though these studies allude to an Arg being important for UDP-Glu activity, studies have found that the specific position of the Arg is not conserved amongst glucuronosyltransferases. Noguchi *et al.* discovered another conserved Arg in P22 of the PSPG motif across Lamiales flavonoid 7-*O*-glucuronosyltransferases (Figure 4) (Noguchi et al., 2009). A multiple sequence alignment revealed that flavonoid 7-*O*-glucosyltransferases had a conserved Trp in P22 of the PSPG motif instead. Swapping Arg for Trp in flavonoid 7-*O*-glucuronosyltransferases UGT88D4, D6, and D7 changed their specificity from UDP-Glu to UDP-Glc. This Arg is also conserved in other glucuronosyltransferases such as UGT88D1 (Nagashima et al., 2000), UGT88D8 (Ono et al., 2010b), and UBGAT (Nagashima et al., 2000). Meanwhile, in VvGT5, an Arg in another position was discovered to be important for UDP-Glu activity (Ono et al., 2010a). Through structural modelling and docking, the authors hypothesised Arg140 (Figure 4) to be interacting with the carboxylic acid of the glucuronic acid moiety. When mutating this residue into a Trp, which is the corresponding residue in glucosyltransferase VvGT1 and galactosyltransferase VvGT6 (Ono et al., 2010a), UDP-Glu activity was abolished while activity with UDP-Glc and -Gal was gained. Interestingly, this Arg is placed in the intersection between a β-sheet and the N5 loop (Section 3) further arguing for the importance of this region.

The observations of these studies suggest the presence of an Arg in the binding site to be vital for UDP-Glu activity. The studies propose the positively charged guanidinium group of Arg to coordinate the negatively charged carboxylic acid of glucuronic acid (pK_a_ of α-d-Glu = 2.93 (Wang et al., 1991)) to stabilise its charge (Osmani et al., 2008; Louveau and Osbourn, 2019). To support this hypothesis, studies have attempted to enable glucuronosyltransferase activity in UGTs with no UDP-Glu specificity by adding an Arg into one of the conserved Arg positions. However, no studies have been able to facilitate UDP-Glu specificity by employing this strategy. UGT88A7 mutant Thr139Ser/Trp367Arg (Noguchi et al., 2009), UGT88E3 mutant Thr150Ser/Trp371Arg (Noguchi et al., 2009), and UGT85B1 mutant Pro26Arg (Osmani et al., 2008) did not confer glucuronosyltransferase activity. These observations could be caused by the fact that for each specific UGT, the exact position of the Arg plays a significant role in whether UDP-Glu activity is enabled. Otherwise, other residues could play a part in correctly coordinating the anomeric carbon with the acceptor substrate or coordinating the carboxylic acid with the Arg. Finally, it is important to mention that glucuronosyltransferases where no Arg can be located close to the sugar moiety of UDP-Glu have also been reported. This includes UGT78H2 (Chen et al., 2021), and GuUGAT (Xu et al., 2016). This demonstrates that there are still unresolved aspects of the structure-function relationship of glucuronosyltransferases. Currently, no crystal structure of a glucuronosyltransferase in a complex with UDP-Glu has been published (Table 1). The elucidation of such a structure would provide valuable insights into the structure-function relationship of glucuronosyltransferases.

## 5 Expanding the donor specificityof UGTs

UGTs that can accept several UDP-sugars serve as the epitome in glycodiversification (Wen et al., 2023; Wu et al., 2023). Hence, it is important to understand which traits lead to UDP-sugar promiscuity. Several natural UGTs accepting several UDP-sugars have been discovered. These UGTs have then been utilised in attempts to either restrict their broad donor substrate scope or use either their structure or sequence information to widen the donor substrate scope of other UGTs. Sb3GT1 from *S. baicalensis* was discovered to accept all five tested donors (UDP-Glc, UDP-Gal, UDP-GlcNAc, UDP-Ara, and UDP-Xyl) (Wang et al., 2019). It was hypothesised that the donor substrate promiscuity was caused by the large active site. To test this hypothesis, the volume of *Sb*3GT1’s active site was decreased by site-directed mutagenesis using the tight active site of VvGT1 as a template. Gly15Ser, Gly15Phe, and Gly15Val all significantly decreased UDP-Gal and UDP-GlcNAc activity which supported the hypothesis of the cavity volume playing a significant role in the donor promiscuity of *Sb*3GT1 (Figure 3D). Similarly, Wen *et al.* tried to alter the donor substrate scope of the promiscuous UGT72AS1 from *Helleborus thibetanus* through site-directed mutagenesis by comparing the sequence information with sequences of both donor promiscuous and donor-specific UGTs (Wen et al., 2023). However, constructed mutants either exhibited conservation of function with all tested donors or a significant decrease with all donor substrates. Only Tyr377Ala (PSPG motif P41) improved activity with UDP-GlcNAc without impacting the function on other donor substrates which could be accredited to reduced steric hindrance.

The same approach has been employed to expand the donor specificity of a UGT with a narrow donor substrate scope (Zhang et al., 2020). This study used the donor substrate-promiscuous GgCGT, which accepts UDP-Glc, -Gal, -Xyl, -Ara, and -GlcNAc, as a template to widen the promiscuity of UGT74AN2 from *Calotropis gigantea,* which could efficiently utilise UDP-Glc and weakly utilise UDP-Gal and -GlcNAc (Huang et al., 2022). This led to the construction of a UGT74AN2 triple mutant with mutations Ile284Arg, Trp390His (PSPG motif P41), and Val391Gly (PSPG motif P42). It was argued that Arg284 would enable a hydrogen bond with C2-OH while His390 and Gly391 would reduce steric hindrance (Figure 3C). Indeed, the triple mutant had improved activity with UDP-Glc, UDP-GlcNAc, and UDP-Gal and gained UDP-Rha activity. The sequence alignment of UGT74AN2 with GgCGT and other donor-promiscuous UGTs showed that a common trait between the donor promiscuous UGTs, which was not observed in the more restricted UGT74AN2, was a non-bulky residue at PSPG motif P42. However, the authors did not test mutating only this position to Ala or Gly. Overall, these studies indicate that active site volume plays an important role in allowing a wide donor substrate scope in UGTs.

## 6 Current state and future directions for elucidating the structure-function relationship governing UGT sugar donor specificity

Based on the described studies, key concepts of the structure-function relationship of UGT sugar donor specificity can be made. First, the PSPG motif plays an integral part in the binding of UDP donors. However, several mutational studies have focused on the terminal residue of the motif leaving the rest of the motif mostly unexplored. While a Gln as the terminal residue seems to be important for UDP-Glc activity, no results have consistently shown that this residue position can be used as a lever to activate and de-activate activity with other UDP-sugars. As demonstrated by several studies (Noguchi et al., 2009; Zhang et al., 2020; Liu S. et al., 2021; Chen et al., 2021), the rest of the motif contains hotspots which are responsible for determining the donor specificity of UGTs and should be further explored. As suggested by Kurze et al., 2022, there could be an interplay between specific positions in the PSPG motif which could be important in the donor specificity of UGTs.

Second, LP1 and LP2 in the N5 loop play a significant role in the donor substrate scope. This is the case for UDP-sugars which differ in the C5/C6 position such as UDP-Glc, -Xyl, and -Rha. Additionally, effects on UDP-Gal, which differs in the C4 position, and on the furanose UDP-apiose, have also been observed. A simple relationship between LP1 and LP2 along with the resulting donor substrate scope could not be described. However, the important factors seem to be the overall polarity along with the flexibility of the loop.

Third, even though discrepancies are observed, an Arg in the binding site of glucuronosyltransferases seems to play an important role in UDP-Glu activity. The Arg potentially serves as a neutraliser of the negatively charged carboxylic acid of UDP-Glu through its positively charged guanidinium group. However, the position of the Arg is not conserved and has, to the best of our knowledge, been described in three separate positions. Solving the structure of a glucuronosyltransferase in complex with UDP-Glu could also help further understand the structure-function relationship and shed light on the binding mode.

Fourth, UGTs with a wide donor substrate scope have mainly been found to possess this trait through a spacious binding site. Mutational studies have supported this theory, but no other theories have currently been tested.

Altogether, the donor substrate scope of UGTs is a complex concept in which several factors seem to play a significant role. The results of these studies can be used as a guiding map to determine which areas and residues to target for altering the donor specificity of UGTs. However, the lack of an in-depth understanding of the structure-function of UGT donor specificity hinders the efficient discovery of UGTs with a specific donor specificity from, e.g., multi-omics data. Studies that have attempted to address and understand this structure-function relationship have mainly resorted to mutational studies adopting a rational engineering mindset Figure 3A. However, studies have shown that residues outside the active site can impact the donor substrate scope of UGTs (Akere et al., 2018). Since no true high-throughput method for assaying UGT activity is currently available, random mutagenesis-based methods, which could help elucidate important areas outside the binding cavity, are currently a time- and material-consuming option. In addition, previous studies have found that the donor substrate scope is also dependent on the acceptor substrate (Wang et al., 2019; Huang et al., 2023), further complicating the situation. Hence, future research efforts and data set curations should also consider the acceptor substrate.

Following these observations, future research efforts could include the development of a true high-throughput UGT activity assay, which would greatly accelerate data generation and unlock a treasure-trove of powerful techniques such as directed evolution and machine learning-based prediction. This would enable this research topic to enter a new paradigm. Moreover, it could be advantageous to establish and strengthen connections between information from diverse organisms and even between various types of carbohydrate-active enzymes (CAZymes) (Quirke and Crich, 2021; Franceus et al., 2022). This could potentially contribute to a more comprehensive understanding of the structure-function relationship, particularly in terms of what determines the specificity of enzymes that share similar substrates. Other research directions could include *in silico* studies such as molecular dynamics simulations with the N5 loop being a potential target of these studies or ancestral sequence reconstruction to uncover a potential evolutionary element of the structure-function relationship (Franceus et al., 2024). Lastly, the emergence of machine learning and explainable artificial intelligence as tools to uncover enzyme mechanisms and structure-function relationships could also prove useful (Taujale et al., 2020; Kouba et al., 2023). Machine learning could allow the exploration of enzyme features outside the binding site and epistatic effects, the combination of donor- and acceptor-substrate features, and the inclusion of UGTs or other CAZymes across organisms.
